# Ovarian reserve in women with sickle cell disease

**DOI:** 10.1371/journal.pone.0213024

**Published:** 2019-02-22

**Authors:** Julia Kopeika, Adeola Oyewo, Sinthiya Punnialingam, Nivedita Reddy, Yacoub Khalaf, Jo Howard, Sofia Mononen, Eugene Oteng-Ntim

**Affiliations:** 1 Assisted Conception Unit, Guy’s and St Thomas NHS Trust, Great Maze Pond, London, United Kingdom; 2 Department of Women & Children’s Health, King’s College London, Guy’s and St Thomas NHS Trust, London, United Kingdom; 3 Haematology department, King's College Hospital, Denmark Hill, London, United Kingdom; 4 Haematology Department, Guy’s and St Thomas NHS Trust, Great Maze Pond, London, United Kingdom; University of the West Indies Faculty of Medical Sciences Mona, JAMAICA

## Abstract

**Background:**

It has been proposed that ovarian sickling and/or iron overload in women with sickle cell disease (SCD) could contribute to gonadal dysfunction, but there are very few published studies. We hypothesised that the above phenomena might impair ovarian reserve.

**Methods:**

A total of 50 SCD patients were case-matched by age, ethnicity, and presence of regular cycles (28±5 days) with 73 patients without a known haemoglobinopathy who required anti-Müllerian hormone (AMH) assessment in a gynaecology clinic. SCD patients had AMH levels taken as part of routine care. The patients were case-controlled and matched with patients who had no haemoglobinopathy in a tertiary centre over a period of one year.

**Results:**

The mean AMH in the SCD case group was 7.6 pmol/l compared with 13.4 pmol/l in the control group (p<0.001). The AMH distributions were subsequently categorised. This showed that SCD patients had a significantly higher chance of having lower AMH in comparison with the control group (OR 2.6 (CI 1.1–6.5, P = 0.02). The proportion of women with AMH > 20 pmol/l was significantly lower in the SCD group (6%) in comparison with the control group (19%) (P = 0.04).

**Conclusions:**

This is the first study showing that women of reproductive age with SCD are more likely to have a low ovarian reserve at a younger age in comparison with patients with no haemoglobinopathy.

## Introduction

Sickle cell disease (SCD) is an autosomal recessive haemoglobin disorder that includes the homozygous (HbSS) genotype and various compound heterozygous genotypes (eg, sickle cell HbSC disease and sickle cell β-thalassemia disease (HbSβ thal). SCD is characterised by a lifelong chronic haemolytic anaemia and intermittent vaso-occlusive pain crises, as well as chronic multi-organ dysfunction including an increased risk of stroke, cardiorespiratory disease, renal failure and infection (due to hyposplenism). It is also associated with a reduced lifespan. In the UK, individuals are identified by the newborn screening programme and medically followed up, allowing for early initiation of infection prophylaxis.

SCD is one of the most common single gene disorders in the world with 300,000 children born with the condition each year [1]. It affects approximately 100,000 people in the United States [2] and 12,500–15,000 in the UK [3]. In 2010, the total UK incidence estimate for SCD was 0.54 cases per 1,000 births, with the highest incidence in South East London (3 per 1,000 births) [4].

Advancements in early detection and management of SCD through national newborn screening programmes in high-income and some middle/low income countries have significantly improved survival and reduced morbidity [5]. As a result of this, life expectancy in individuals with SCD has risen markedly since the 1970s, from their teenage years to well into their fifth decade in most countries [6,7]. This has prompted a paradigm shift in providing care for patients with SCD from not only ensuring survival, but also improving quality of life and reproductive potential.

SCD is associated with gonadal dysfunction in men, with reports of both primary and secondary hypogonadism described in literature [8–10]. However, very little is known of its effect on gonadal function in females [7]. Early studies demonstrated a delay in sexual development in females with SCD, including delay in menarche [11]. It has been suggested that chronic transfusion and iron overload in patients with severe manifestations of SCD may also be associated with impaired gonadal function [12]. It has also been hypothesised that frequent episodes of intravascular sickling, vessel occlusion, infarction, as well as tissue hypoxia associated with chronic anaemia, could account for the ovarian dysgenesis and premature ovarian failure in women with SCD [13]. Accurate assessment of ovarian reserve can be helpful in predicting fertility life span and potential [14, 15]. Ovarian reserve can be assessed in several ways, including by assessment of reproductive hormones (FSH, LH, oestradiol and anti-Müllerian hormone (AMH)) and antral follicular count with ultrasound [16].

AMH has emerged as a useful marker for assessment of ovarian function [16, 17]. It is produced by the granulosa cells of pre-antral and small antral follicles in the ovaries during the reproductive years and its level can be measured in serum [18]. AMH is solely produced in the growing ovarian follicles and does not show clinically relevant changes across the menstrual cycle [19]. Investigation of the effects of treatment regimens used in SCD on gonadal function show that AMH levels were low in peripubertal patients with SCD who had undergone bone marrow transplantation and in patients who had been treated with gonadotoxic agents such as hydroxycarbamide [20]. This is not surprising, as common preparation for bone marrow transplant involves exposure to gonadotoxic alkylating agents such as cyclophosphamide and busulfan. To date there are no studies investigating whether SCD *per se* causes any impairment in ovarian function. The objective of this study was to assess the ovarian reserve in women with SCD by evaluating AMH levels and comparing them to matched controls.

## Materials and methods

### Patient/cohort characteristics

As part of a service evaluation, the AMH levels in female patients with SCD were analysed. Sequential patients aged between 25–45 with no actively reported concern about their fertility or menstrual cycle who attended the haematology clinic at Guy’s and St Thomas NHS Foundation Trust in 2011 were included in the analysis. At the time of recruitment, the patients were in a steady state with no reported acute symptoms or pain requiring hospitalisation. Exclusion criteria included previous bone marrow transplant, current pregnancy, and breastfeeding. Patients of all SCD genotypes were included in the analysis. These results were subsequently compared with results from a matched control patient population who had either AA or AS genotypes, and attended the reproductive medicine clinic due to male, tubal, or unexplained factor subfertility. All matched case controls had regular menstrual cycles. The subjects and controls were matched by age and ethnicity. More than one control per subject was used where possible. Serum samples were collected at any time of the menstrual cycle from the SCD patients during their routine annual visit to the haematology clinic, and from the controls as part of the routine assisted conception work-up. All individual patient serum AMH measurements were performed in the chemical laboratories of Guy’s and St Thomas NHS Foundation Trust. AMH assay was performed using the AMH Generation II ELISA assay. Based on hormone results, patients’ serum AMH levels were classed as—negligible: < 1.5 pmol/l; reduced: 1.5 to 6.5pmol/l; normal 6.6 to 19.8 pmol/l; high: > 19.8pmol/l. The lowest detection limit and the intra and inter-assay coefficients of variation were 0.57 pmol/l, 4% and 5.5% respectively.

### Statistical analysis

A power calculation previously done by Schram et al (2015) suggested that at least 20 subjects and 20 control patients matched by age would provide 82% power to detect a clinically relevant mean difference in AMH of 4 pmol/L between the groups [21]. This assumption was made based on a common AMH standard deviation of 5 pmol/L [22], a correlation of 0.3 between members of the pairs due to matching, and a significance level of 0.05 using a two-sided paired t-test. We had twice as many cases in this study, which allowed us to have sufficient power to establish the difference based on the above assumptions.

Differences between the two cohorts and across the AMH groups within each cohort were tested using the Mann-Whitney test for continuous data and the **χ**2 test for categorical data. For all analyses, p <0.05 was considered statistically significant. Logistic regression analysis was used to predict the odds ratio for having low ovarian reserve adjusted to age and hydroxyurea. Statistical analysis was performed using SPSS 19.0 software.

## Results

A total of 50 SCD patients were compared to 73 controls of the same age (range 25 to 45) and ethnicity. The mean and median ages in case and control groups were 35.1 and 35 vs 35.8 and 36 years respectively. There was no statistical difference in age between these two groups (P = 0.43, Mann-Whitney test).

The demographic data is outlined in Table 1. The majority of SCD patients had haemoglobin SS (78%) and the majority of the control group had haemoglobin AA (77%). 16% of the SCD cohort were taking hydroxycarbamide. Chronic lung disease (evident from previous history and radiographic features of sickle cell chronic lung disease) was seen in one fifth of SCD patients included in this study. 38% of women in the control group had tubal factors as an indication for IVF. The rest had either male, unexplained, or other factor subfertility. Before we included trait patients in the control group, we checked if there was any difference in AMH levels between patients with HbAA and other non-SCD patients. This was proven to be non-significant (P = 0.9). Therefore, trait patients were not excluded from the overall control group.

**Table 1:** Background characteristics of patients with and without SCD.

	Control n = 73	SCD n = 50
**Age**		
Mean, SD	35.8 ± 4.8	35.1 ± 5.9
Range	25–45	25–45
**Ethnicity**		
Black	100%	100%
**Hb status**		
AA	77%	
AS	16%	
Other traits	7%	
SS		78%
SC		18%
Other sickle		4%
**Patients on hydroxycarbamide**	Not applicable	16%
**Chronic lung disease**		20%
**Non-smoker**	94%	98%
**Indication for IVF:**		Not applicable
Tubal	38%	
Male	20%	
Unexplained	20%	
Other	23%	

**Fig 1:**
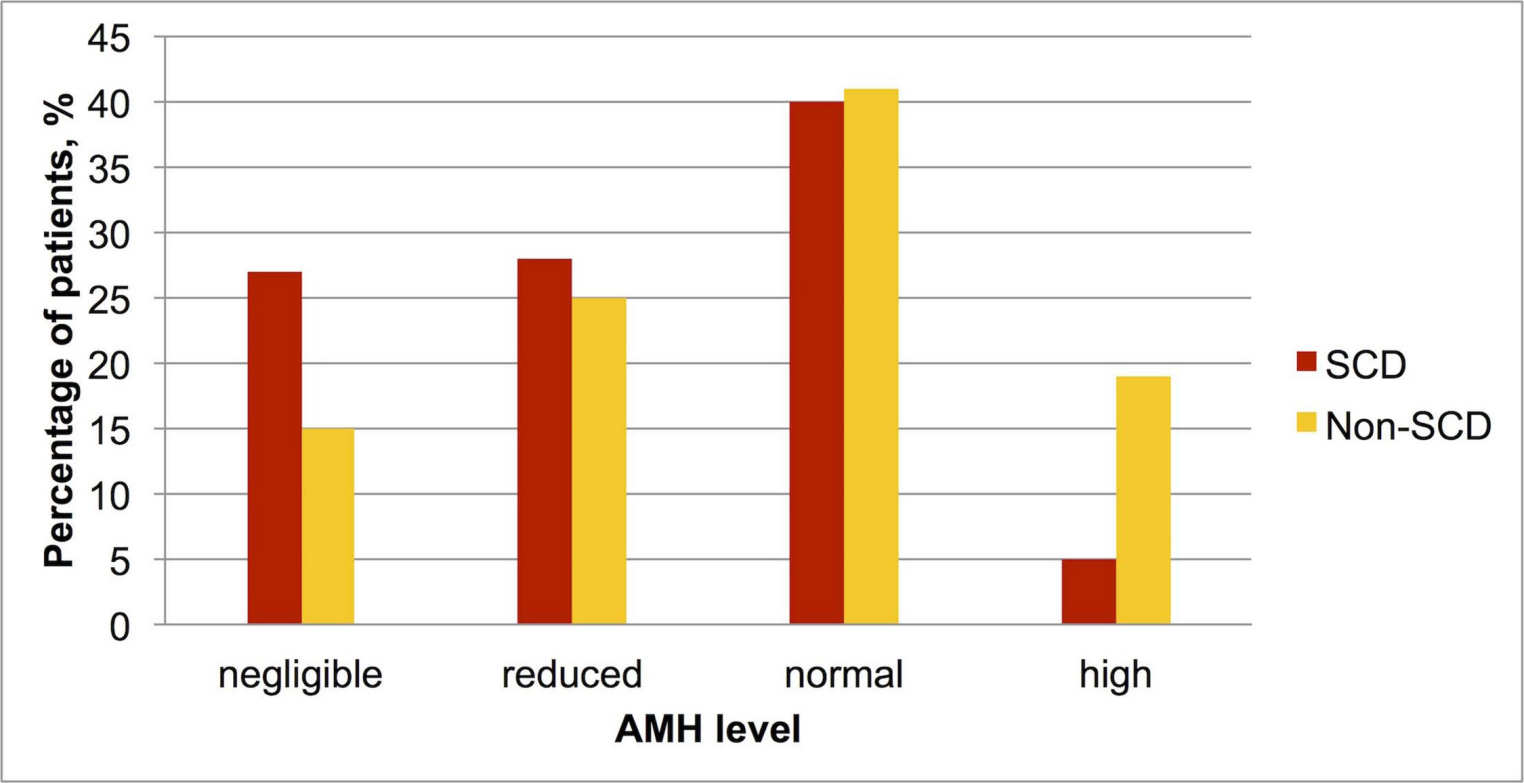
Distribution of different AMH categories in patients with and without SCD. AMH categories: negligible–less than 1.5 pmol/l; reduced -1.5 to 6.5 pmol/l; normal- 6.6 to 19.8 pmol/l; high–above 19.8 pmol/l.

Comparison of AMH levels between the two groups showed that patients with SCD had significantly lower levels of AMH in comparison to the controls (7.6 vs 13.4pmol/l, p = 0.01) using the Mann-Whitney test (Table 2). Numbers of patients and controls with AMH levels in each category (negligible, reduced, normal or high) were subsequently compared (Fig 1). Logistic regression analysis showed that SCD patients had a significantly higher chance of having low AMH in comparison with the control group (OR 2.6 (CI 1.1–6, P = 0.02)). This was adjusted to age and hydroxyurea. The proportion of women with high AMH levels (more than 20 pmol/l) was also significantly lower in the SCD group (6%) in comparison with the control group (19%), P = 0.04.

**Table 2:** AMH levels in women with and without SCD.

AMH	Control	SCD	P value
**mean± SD, pmol/l**	13.4± 14.4	7.6± 7.8	P = 0.01*
**median**	8.4	4.9	
**25**^**th**^ **centile**	2.5	1.1	
**50**^**th**^ **centile**	8.4	4.9	
**75**^**th**^ **centile**	17.7	10.8	

*Adjusted to hydroxyurea

**Fig 2:**
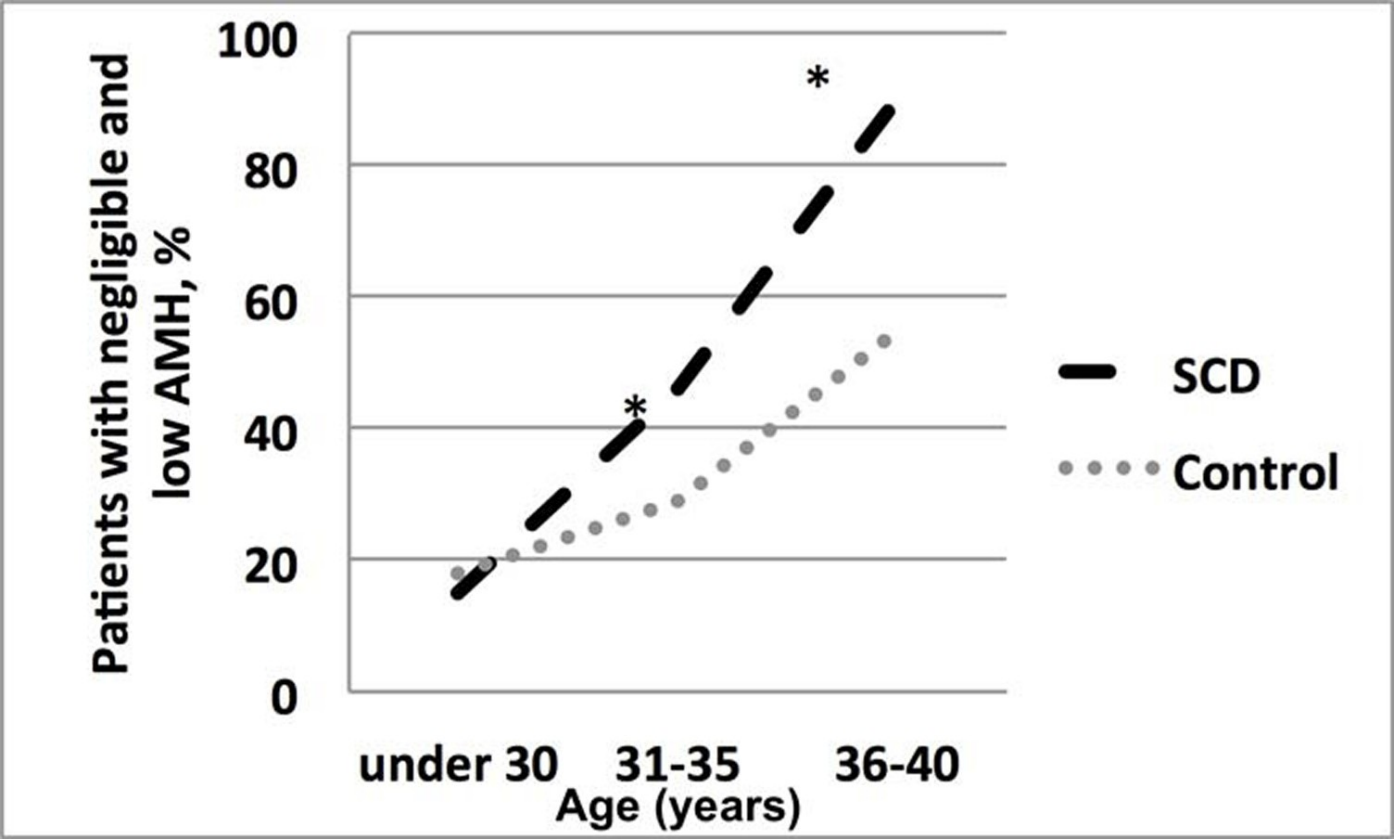
Prevalence of patients with negligible (<1.5 pmol/l) and reduced (1.5–6.5 pmol/l) AMH among SCD and control patients (* p≤0.5).

**Fig 3:**
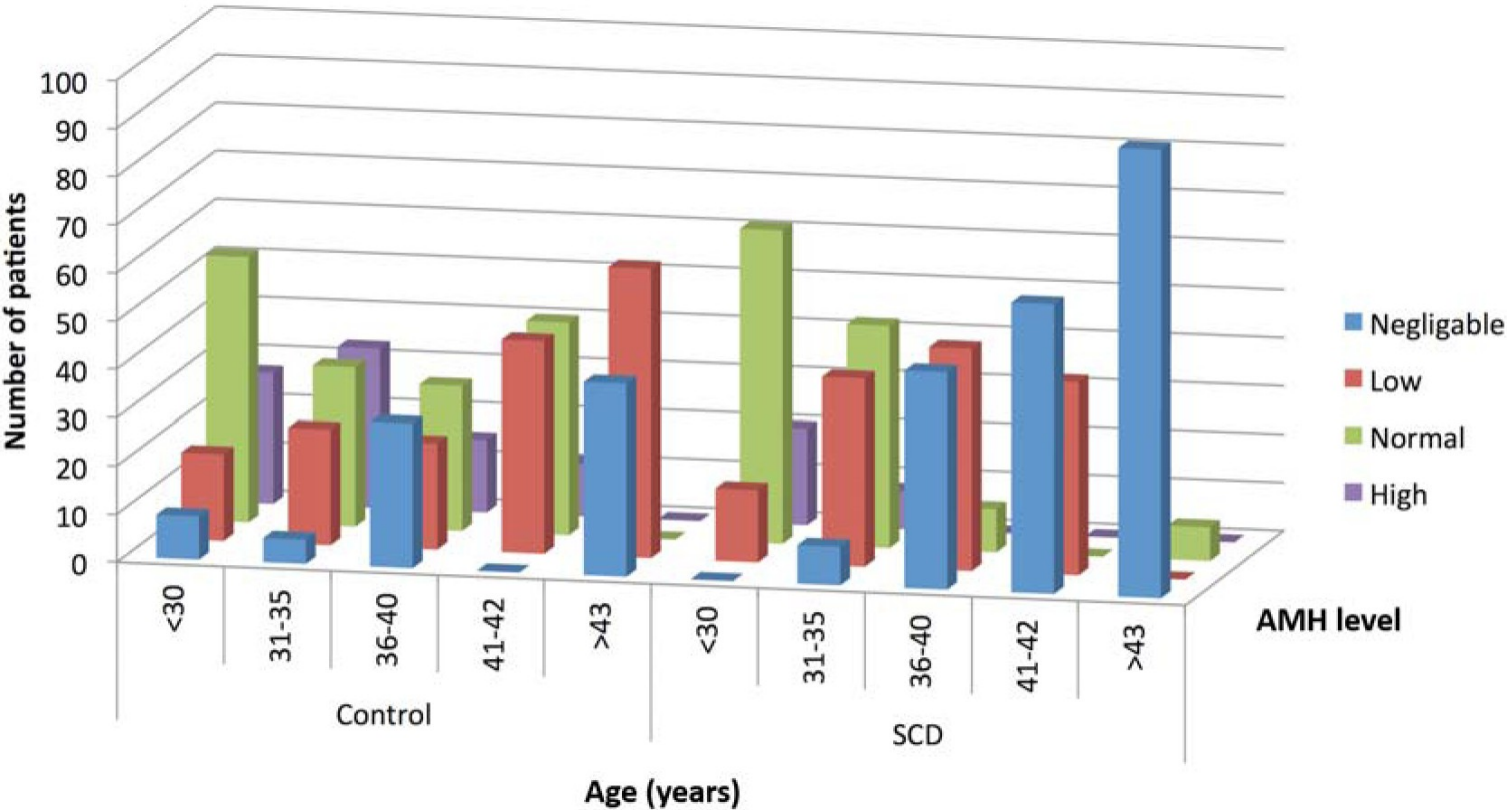
Distribution of different AMH categories in different age groups in patients with and without SCD.

As expected, the prevalence of low (negligible or reduced) AMH levels increased with age (Fig 2), however this was identified at a much earlier age in the SCD group. In the 36–40 year age group, 55% of the control group had low or negligible AMH levels, compared with 90% of the SCD group (p = 0.043) (Fig 2). In patients <30 years, there was no significant difference in the prevalence of patients with normal AMH levels in the control and SCD group (55% vs 65%, p = 0.59) (Fig 3).

We tried to see if the mean AMH levels for SCD patients with or without pulmonary disease in the current study were different. It was found to be 5.76±6 and 8.6±8.4, respectively. This did not reach statistical significance (P = 0.2). However, the total number of patients with chronic pulmonary disease was only 10 in the current study.

## Discussion

This report is the first to show that women with SCD have a significantly greater incidence of negligible or low levels of AMH, when compared to age-matched controls without haemoglobinopathy. This is due to decreased ovarian reserve in patients with SCD. Serum AMH level has been shown to decline throughout a woman’s reproductive lifespan [14]; our study showed that it declines faster in women with SCD than in a control population from the age of 30 (Fig 2).

One may argue that the above difference was due to the fact that the control cohort in this study had an unusually high number of patients with high AMH levels. Higher AMH levels are known to occur at a younger age and in women with polycystic ovarian syndrome (PCOS), but there was no difference in age between the groups and none of the patients included in the control group had PCOS. The prevalence of high level AMH in our cohorts is less than that previously reported for the normal population (18.7%) [23], therefore there is no evidence that the chosen control population had an over-representation of patients with high AMH levels. The control group in this study were patients undergoing assisted conception: this choice of patient population could be supported by the evidence suggesting that there is no significant difference in prevalence of low AMH between IVF patients and women with no reported fertility problems [24].

It is important to consider other variables that are known to affect AMH such as ethnicity, smoking, body mass index (BMI), and usage of combined oral contraception [19, 25–27]. Studies have shown significantly lower levels of serum AMH in black women in comparison to white women, as well as significantly greater age-related decline in AMH over time compared with white women [26, 28, 29]. Both the subjects and control population in our study were from the same ethnic background, which might imply an even greater predisposition to reduced ovarian reserve in the SCD population. This is especially important considering that SCD is particularly prevalent in a population with significantly lower AMH levels compared to others.

There was no significant difference between prevalence of smokers in the studied cohorts (P = 0.29%), therefore the decline in AMH levels observed in our study group could not be attributed to the impact of smoking hence minimizing this confounding factor.

It has been shown previously that AMH levels were significantly lower in obese women with BMI greater than or equal to 30 kg/m^2^ compared to non-obese women [30–32]. Our control population had a mean BMI of 26.5±3.6 with only 3 (4%) patients having BMI>30. Patients with SCD tend to have low or normal BMI due to chronic disease [33]. A recent study completed in USA including 100 SCD patients with a similar mean age reported a mean BMI of 26.3±6 [34].

The effect of oral contraceptive pills on AMH remains controversial, with some studies showing no effect [35, 36] while others demonstrate a reduction [37, 38]. However, a more recent large population-based study has convincingly demonstrated ≤ 20% reduction in AMH in long-term users of combined oral pills (COP) [28, 37]. Therefore, it would be important to consider the possible influence of COP on the data obtained in the current study. The control group did not use COP, as this population had been actively trying to conceive for at least one year. The information on current usage of contraception in the case cohort is not available. Although COP is relatively contraindicated for patients with SCD, some of these patients might still be on this form of contraception. Even under the assumption that the SCD patients in the studied cohort were on COP, the mean AMH level increased by 20% in the SCD group, which would still be statistically lower than the AMH level in the control group.

We would also like to put forward the possible explanation for finding reduced ovarian reserve in patients with SCD. We continue to speculate, in line with the hypothesis proposed by some of the authors of this earlier paper [13], that chronic sickling and vessel occlusion could account for hypoxia of different tissues, including ovaries. Repeated ovarian hypoxia, in turn, could be detrimental for follicle pool [39]. This could cause depletion of ovarian reserve faster than in cases with no chronic hypoxic insult to ovarian tissues. The mean AMH for SCD patients with or without pulmonary disease in the current study was not statistically significant (P = 0.2). However, the total number of patients with pulmonary disease was low (n = 10). In the future it would be interesting to investigate if the severity of the disease has an impact on ovarian function.

Until now, there are no documented studies specifically examining AMH levels as a biomarker of ovarian reserve in women with SCD. In the current study, a small proportion of patients were on hydroxycarbamide. However their mean AMH (8.8 ± 8.3) was not significantly different from patients not taking this medication (P = 0.8), therefore we did not exclude them from our analysis.

There are some limitations to this study, which need to be recognised. Firstly, it has a relatively small cohort of patients with SCD and not all of them have exactly the same genotype and severity of disease. However, since the majority of patients had Hb SS, this may suggest that the overall data could be applicable for this population of patients. Some of the variables that have the potential to reduce AMH level, such as COP and BMI, were extrapolated in this cohort rather than established with certainty. Further, our control for comparison was not derived from the “general” population of people, but from patients attending a gynaecology clinic. Current literature shows that in a white population, there was no difference in AMH between “normal-fertile” and an infertile population of women [25]. Therefore, we believe that the current control cohort was an adequate choice since the primary outcome of this study was AMH, rather than fertility outcome.

There are also several merits of this study. Firstly, it is the first cross-sectional study to compare ovarian reserve in patients with SCD with no reported fertility issues. Secondly, even though the total number of patients is relatively low, it has sufficient power to establish significant difference. Thirdly, the assessment of AMH was done in the same laboratory over the same period of time.

The clinical implications of this study may be far-reaching for women with SCD. Whilst there is a current trend of delaying child-bearing among the population in Western societies [40,41] with increasing incidence of first-time mothers over the age of 35 [42,43], women with SCD need to be aware that significantly lower AMH levels may mean having reduced ovarian reserve and hence a relatively shorter reproductive lifespan.

Further studies are required to establish potential means of preventing accelerated ovarian aging in women with SCD. It would be important to look at long-term data to see if the severity, genotype of SCD, and frequency of acute sickling episodes have an accelerative harmful influence on ovarian tissue.

## Conclusion

In conclusion, to the best of our knowledge our study is the first to show that women of reproductive age with SCD have significantly lower AMH levels, which may be indicative of reduced ovarian reserve when compared to healthy controls. Further studies in women with SCD to assess ovarian function and its impact on reproduction are needed in order to ensure that these patients are adequately supported and informed of reproductive choices that best suit them.

## Supporting information

S1 Dataset Full dataset used in the study including ages, BMI, smoking and AMH levels of patients with SCD and without SCD. (XLSX)
